# Systems proteogenomics for precision oncology

**DOI:** 10.18632/oncotarget.26601

**Published:** 2019-01-22

**Authors:** Frederick Klauschen

**Affiliations:** Frederick Klauschen: Systems Pathology Group, Institute of Pathology, Charité Universitätsmedizin Berlin, Germany; German Cancer Consortium, Berlin Partner Site and German Cancer Research Center, Heidelberg, Germany

**Keywords:** systems medicine, proteomics, genomics, precision oncology, tumor profiling

Genomics and particularly mutational profiling have deepened our understanding of cancer pathology and laid the foundation for precision therapies as a major field in modern oncology. Comprehensive genetic profiling has even led to proposals to replace the current histology-based WHO tumor typing with molecular classes based on the rationale that similar molecular alterations have similar clinical relevance across cancers [1-3]. However, targeted drugs against oncogenic mutations shown to be efficacious in one cancer are often ineffective in another [4]. But not only is it often not possible to transfer druggability of a mutation from one histological tumor type to another, response to targeted therapies shows significant variations also within the same tumor type. While some patients show long-term benefit, others quickly relapse or show no response to therapy despite identical actionable mutations. The reasons for this variability in biological behavior and therefore clinical relevance lie in the often complex genetic background in many tumors, but are also related to epigenetic, post-transcriptional and post-translational modifications not captured by mutational profiling alone. Non-small cell lung cancer, for instance, harbors over 800 genetic aberrations [5], on average, and although many of those are believed to be passenger mutations, a substantial number of mutations can be expected to be involved in the modulation of resistance mechanisms. However, the influence of these rare “tail” mutations is difficult to evaluate for two related reasons. First, even if a large number of mutations can be excluded based on prior biological knowledge, identifying drug combinations to overcome resistance is challenging even for a small number of mutations due to the combinatorial complexity (as an example, 780 alternative two-drug combinations exist for 40 targets [6]). Moreover, even if candidate targeted combination therapies are identified, patient recruitment for clinical trials is difficult due to the rarity of the druggable mutations. Therefore, ways beyond genomics have to be found to identify functionally and clinically relevant molecular alterations within the complex mutational landscape of cancer.

**Figure 1:**
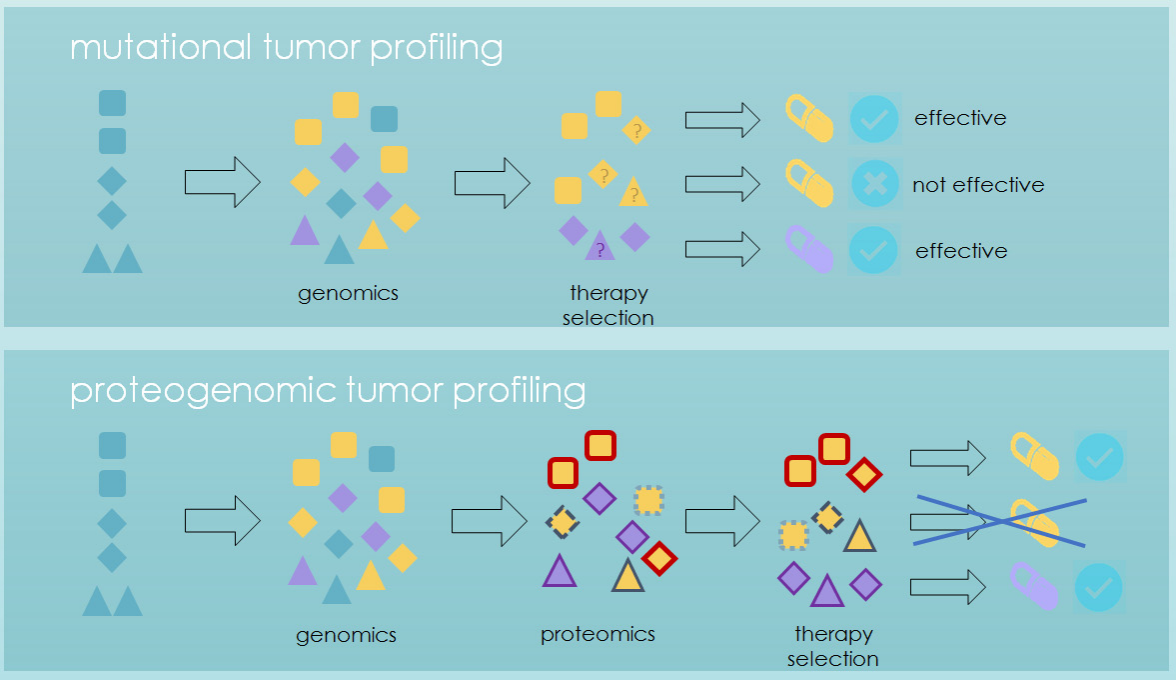
Proteomics-augmented mutational tumor profiling Top: Current prediction of the efficacy of targeted therapies is mainly based on mutational tumor profiling. Druggability of mutations is established for histological tumor types (shapes) and while some mutations (fill colors) can be targeted across cancers, the efficacy of a drug established in one tumor type is not generally transferable to another tumor type. Also, some patients do not respond to targeted therapy despite the right mutation-histotype-combination. Bottom: To better predict the druggability of mutations within and across cancer types, mutational profiling can be complemented by clinical proteomics, which uncovers distinct proteomic profiles (outline colors) for mutations with functional and clinical relevance, reduces the complexity of the mutational landscape and identifies targets for combination therapies.

One way to assess the functional impact of genetic alterations in cancer is to study their relationship with corresponding (phospho-)proteomic profiles. Using publicly available proteogenomic profiling data across major cancer types, we propose a computational approach that systematically evaluates if and to what extent genetic aberrations observed in tumors are associated with distinct proteomic profiles [7]. Our analysis shows that distinct proteomic profiles are observed for mutated vs. wildtype genes in cancers known to be druggable for the respective mutations, which is not the case for mutations for which targeted therapies are ineffective. This approach therefore facilitates predicting potentially oncogenic and/or actionable genes in the context of different histological tumor types. While this shows the capacity of proteomics to complement genomic profiling with respect to assessing functional impact for gene mutations with sufficient frequencies to allow for computational evaluation, the ultimate goal of such approaches must be the prediction on the level of individual patients to support precision oncology.

To achieve this, we further propose a combined experimental and computational systems proteogenomics approach that allows for a reduction of the mutational complexity and facilitates the identification of functionally relevant molecular aberrations that can be exploited to overcome resistance against targeted (mono-)therapy in individual patients [8]. It combines mutational profiling with proteomics and uses experimental perturbation with targeted drugs against which resistance has developed. Cell or tissue culture models are treated with the respective inhibitors subsequent to which time-course discovery phosphoproteomics is performed in comparison to a standard. Because static proteomics data is similarly complex as the mutational profiles, they do not per se facilitate a better understanding of the pathological mechanisms, but dynamic changes in protein phosphorylation and expression analyzed after perturbation and compared with the t=0 status may offer a more specific picture. This is supported by bioinformatic methods including differential correlation [9], which helps identify relevant groups of phosphoproteins, as well as network models used to topologically relate mutational and proteomic profiles. Through these data analysis and integration steps, the complexity of the molecular profiling data can be substantially reduced to a short list of likely functionally relevant target molecules that can then be further evaluated experimentally and clinically.

In summary, the ability to interpret and assess the functional and clinical relevance of the increasingly comprehensive mutational profiling data accruing not only in research but also in clinical cancer diagnostics is reaching its limits when relying only on pre-clinical experimental data and knowledge on biological pathways. Moreover, the combinatorial complexity of potential druggable target combinations is incompatible with testing in classical clinical trials. As a solution, combining genomic with proteomic profiling and classical computational analysis as well as advanced machine learning techniques can contribute to identifying functionally and clinically relevant molecular alterations which in the future may complement molecular diagnostics for precision oncology.
